# Increased oxidative phosphorylation in response to acute and chronic DNA damage

**DOI:** 10.1038/npjamd.2016.22

**Published:** 2016-10-13

**Authors:** Lear E Brace, Sarah C Vose, Kristopher Stanya, Rose M Gathungu, Vasant R Marur, Alban Longchamp, Humberto Treviño-Villarreal, Pedro Mejia, Dorathy Vargas, Karen Inouye, Roderick T Bronson, Chih-Hao Lee, Edward Neilan, Bruce S Kristal, James R Mitchell

**Affiliations:** 1 Department of Genetics and Complex Diseases, Harvard T. H. Chan School of Public Health, Boston, MA, USA; 2 Division of Environmental Health, Vermont Department of Health, Burlington, VT, USA; 3 Department of Neurosurgery, Harvard Medical School, Brigham and Women’s Hospital, Boston, MA, USA; 4 Rodent Histopathology Core, Department of Pathology, Harvard Medical School, Boston, MA, USA; 5 Genetics and Metabolism Division, Boston Children's Hospital, Harvard Medical School, Boston, MA, USA

## Abstract

Accumulation of DNA damage is intricately linked to aging, aging-related diseases and progeroid syndromes such as Cockayne syndrome (CS). Free radicals from endogenous oxidative energy metabolism can damage DNA, however the potential of acute or chronic DNA damage to modulate cellular and/or organismal energy metabolism remains largely unexplored. We modeled chronic endogenous genotoxic stress using a DNA repair-deficient *Csa*^*−/−*^*|Xpa*^*−/−*^ mouse model of CS. Exogenous genotoxic stress was modeled in mice *in vivo* and primary cells *in vitro* treated with different genotoxins giving rise to diverse spectrums of lesions, including ultraviolet radiation, intrastrand crosslinking agents and ionizing radiation. Both chronic endogenous and acute exogenous genotoxic stress increased mitochondrial fatty acid oxidation (FAO) on the organismal level, manifested by increased oxygen consumption, reduced respiratory exchange ratio, progressive adipose loss and increased FAO in tissues *ex vivo*. In multiple primary cell types, the metabolic response to different genotoxins manifested as a cell-autonomous increase in oxidative phosphorylation (OXPHOS) subsequent to a transient decline in steady-state NAD+ and ATP levels, and required the DNA damage sensor PARP-1 and energy-sensing kinase AMPK. We conclude that increased FAO/OXPHOS is a general, beneficial, adaptive response to DNA damage on cellular and organismal levels, illustrating a fundamental link between genotoxic stress and energy metabolism driven by the energetic cost of DNA damage. Our study points to therapeutic opportunities to mitigate detrimental effects of DNA damage on primary cells in the context of radio/chemotherapy or progeroid syndromes.

## Introduction

The process of generating energy through mitochondrial oxidative phosphorylation (OXPHOS) inevitably results in production of free radicals that can damage cellular macromolecules, including DNA. Consistent with this, interventions that increase OXPHOS (e.g., fasting) can also lead to an increase in free radical generation, at least transiently.^1^ Because of the potential detrimental effects of free radical damage, cells have evolved a variety of mechanisms of detoxification, many of which respond to oxidative stress in an inducible fashion. For example, the transcription factor NRF2 controlling expression of key enzymes in the glutathione pathway is itself activated by oxidative and electrophilic stressors.^2^ On the other hand, because energy generation through aerobic glycolysis rather than OXPHOS could potentially limit further oxidative DNA damage accumulation,^3^ altering metabolism could be another potential strategy to limit free radical damage. As proof of principle, a number of cell types can rapidly shift between OXPHOS and glycolysis depending on environmental cues, e.g., upon antigen stimulation in the case of immune cells,^4^ although the potential of DNA damage to contribute to this process remains untested.

DNA damage can also be energetically costly, e.g., upon activation of the DNA damage response protein, PARP-1. PARP-1 binds directly to single-stranded DNA and generates chains of poly-ADP-ribose (PAR) in response to breaks that occur directly or indirectly upon ultraviolet radiation (UV),^5^ ionizing radiation (IR),^6^ topoisomerase inhibition^7^ or DNA alkylation.^8^ PARP-1 activation by massive DNA damage leads directly to NAD+ depletion and subsequently to ATP depletion and cell death via necrosis^9^ or apoptosis.^10^ Although this demonstrates the indirect potential of DNA damage to alter cellular energy levels, with profound downstream consequences on cell fate,^11^ the molecular mechanisms underlying PARP-1 induced ATP loss remain unresolved.^12^ One possibility is through depletion of NAD+, a key metabolite in both glycolytic and oxidative energy metabolism.

Although the PARP-1 example serves to illustrate the high energetic cost of excessive DNA damage, how cells respond to perturbations in energy homeostasis induced by sublethal doses of DNA damage remains poorly characterized. One clue is the discovery that genotoxic stressors including UV-C, IR^13^ and cisplatin^14^ can activate the AMP-activated protein kinase (AMPK), which acts to restore energy balance by inhibiting ATP-consuming processes and activating processes to generate ATP.^15^ AMPK is regulated allosterically by AMP, but also requires phosphorylation by upstream kinases such as LKB1 and CaMKKβ^15^ or p53 transcriptional targets, sestrin 1/2.^16^ Currently, neither the mechanism by which DNA damage activates AMPK, nor the consequences of such activation, are clearly understood.

Thus, although it is clear that the byproducts of normal energy metabolism can cause DNA damage, and that DNA damage can impact cellular energy metabolism, whether and how DNA damage itself exerts feedback control over energy metabolism is poorly characterized, particularly on the organismal level. Here, we set out to directly test the potential of DNA damage to affect energy metabolism, and to identify common adaptive metabolic changes on the cellular and organismal levels in response to both chronic and acute DNA damage.

For chronic damage, we utilized a mouse model of the congenital DNA repair deficiency disorder, Cockayne syndrome (CS). CS is characterized by growth failure, loss of adiposity, neurodegeneration and photosensitivity without prevalence of skin cancer.^17^ It is caused by alterations in proteins including CSA or CSB that share a common function in transcription-coupled nucleotide excision repair (NER) of bulky, helix-distorting lesions, e.g., those caused by UV light that sterically hinder RNA polymerases and block transcription.^18^ Recently, we reported a mouse model of CS lacking both *Csa* and *Xpa,* another NER gene, (heretofore referred to as CX) that recapitulates many aspects of the human progeria.^19^ As a model of acute genotoxic stress, we subjected wild-type (WT) mice and cells with genotoxins (UV-C, IR, mitomycin C (MMC) and irinotecan) giving rise to diverse spectrums of DNA lesions. Here, we report that multiple forms of acute and chronic genotoxic stress engender a PARP-1-dependent metabolic switch characterized by increased fatty acid oxidation (FAO) and OXPHOS as an adaptive response to maintain energy homeostasis on both organismal and cell-autonomous levels.

## Results

### Perturbations in energy metabolism indicative of improved metabolic fitness in CX mice

**Figure 1:**
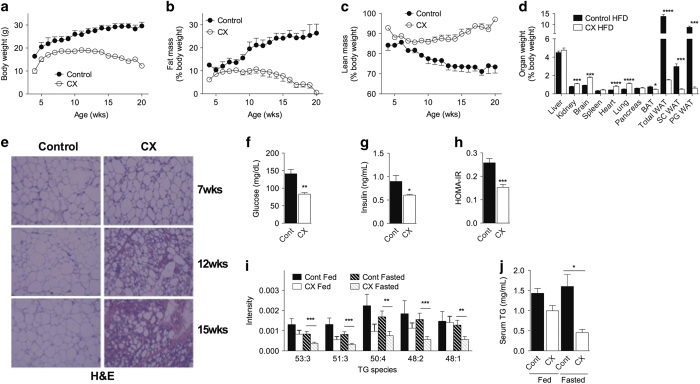
Perturbations in energy metabolism indicative of improved metabolic fitness in CX mice. (**a**–**c**) Changes in body weight (**a**), percentage fat (**b**) and lean mass (**c**) over time in CX and control mice (*n*=14–16/group). (**d**) Organ weights upon harvest of 14 week mice on a high-fat diet (HFD) expressed as a percentage body weight, *n*=4/group; Student’s *t*-test. (**e**) Hematoxylin and eosin (H&E) staining of paraffin-embedded perigonadal white adipose tissue (WAT) from control and CX mice. (**f**–**h**) Blood glucose (**f**), serum insulin levels (**g**) and HOMA-IR (**h**) of 12–16 week control and CX mice (*n*=7–11/genotype); Student’s *t-*test. (**i**) Relative intensity of the indicated triglyceride (TG) species in serum from fed or fasted CX or control mice by unbiased lipidomics (CX=5/group, control=10/group); two-way analysis of variance (ANOVA) with Bonferroni post-test. (**j**) Total serum TG in fasted and fed WT and CX mice (*n*=3/group); Student’s *t-*test. *P<0.05, **P<0.01, ***P<0.001, ****P<0.0001.

A number of mouse models with severe symptoms of CS (‘NER progeria’ in mice), display apparent alterations in systemic metabolism during post-natal development, including loss of adiposity, perturbations of the post-natal GH-IGF-1 axis and hypoglycemia contributing to death before weaning.^20^ Using the CX mouse model that survives weaning with high penetrance,^19^ we first characterized changes in adiposity, glucose and lipid homeostasis as markers of whole-body energy metabolism. Although physically smaller than control (includes WT, CSA single KO and XPA single KO) mice, CX mice increased in body mass during post-natal development at a similar rate. Maximal body mass was attained by 8 weeks and was stable for ~5 weeks before onset of terminal senescent weight loss over the remainder of the lifespan (Figure 1a). Body fat in CX mice calculated as either a percentage of body weight (Figure 1b) or in absolute grams (Supplementary Figure S1a) paralleled body weight, peaking ~8 weeks and declining after 13 weeks. Although the absolute lean mass declined with age in CX mice (Supplementary Figure S1b), the percentage lean mass remained stable between 13 and 20 weeks (Figure 1c). CX mice fed a high-fat diet (HFD) failed to normalize relative and absolute weights of subcutaneous or perigonadal white adipose tissue (WAT) depots compared with controls (Figure 1d, Supplementary Figure S1c), consistent with the failure of HFD feeding to rescue CX longevity.^19^ Progressive histological investigation of WAT from CX mice showed a decline in mature adipocyte size (Figure 1e), but no visible evidence of crown-like structures indicative of inflammatory macrophage activation by hematoxylin and eosin or in WAT from 16 week CX mice as shown by F4/80 immunohistochemistry (Supplementary Figure S1d) or fluorescence-activated cell sorting (FACS) analysis of F4/80+/CD11b+ stromal vascular fraction (Supplementary Figure S1e). These data suggest altered functionality of CX WAT rather than loss of cellularity due to inflammation.

In 14–16 week CX mice, fasted blood glucose and insulin levels were significantly reduced (Figure 1f,g), leading to an improved HOMA-IR (Figure 1h). Insulin challenge trended towards increased glucose disposal in 14 week CX mice (Supplementary Figure S1f), and glucose tolerance tests revealed no difference (Supplementary Figure S1g), suggestive of improved whole-body insulin sensitivity but reduced capacity for stimulated insulin secretion in CX mice, similar to long-lived models such as growth hormone receptor knockout mice.^21^ Circulating leptin levels in the fasted state were significantly decreased in CX versus control mice (Supplementary Figure S1h) but adiponectin levels were unchanged (Supplementary Figure S1i). Analysis of serum lipids using unbiased mass spectrometry revealed significant differences in multiple lipid species in the fasted state and a trend towards a reduction in the fed state in 16 week CX mice relative to both WT and single KO controls, including a number of individual triglyceride species (Figure 1i, Supplementary Figure S1j). Measurement of bulk serum triglyceride in a separate cohort of control and CX mice confirmed a significant reduction in the fasted state (Figure 1j).

### Increased FAO in CX mice *in vivo* and cells *in vitro*

**Figure 2:**
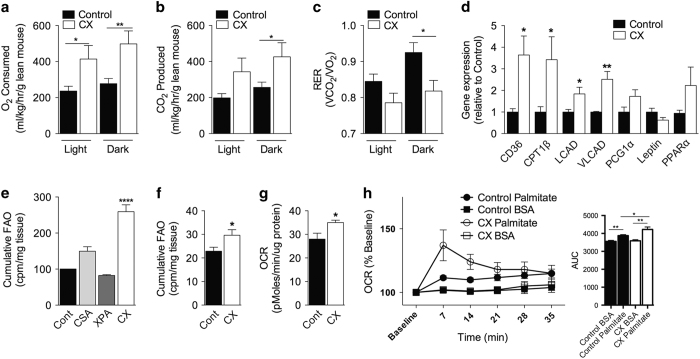
Increased fatty acid oxidation (FAO) in CX mice *in vivo* and in cells *in vitro.* (**a**–**c**) Indirect calorimetry of 8–14 week control and CX mice (*n*=12/group) showing O_2_ consumed (**a**), CO_2_ produced (**b**) and respiratory exchange ratio (RER) (**c**) all corrected by lean body mass; Student’s *t-*test. (**d**) Gene expression of 12–16 week control and CX muscle (*n*=6/group); Student’s *t-*test. (**e**) Cumulative FAO of tritiated palmitate of soleus muscle from 16 week mice (*n*=5–8/group); 1-way analysis of variance (ANOVA) with Dunnett’s post-test. (**f**) Cumulative FAO of tritiated palmitate of liver of 16 week mice (*n*=5–8/group); Student’s *t-*test. (**g**) Basal oxygen consumption rate (OCR) of mouse dermal fibroblasts (MDFs) measured by Seahorse cell metabolism analyzer in three independent lines of each genotype; Student’s *t-*test. (**h**) OCR expressed as a percentage of baseline within cell line of control and CX MDFs by Seahorse after addition of 200 μmol/l bovine serum albumin (BSA)-conjugated Palmitate with area under the curve anaylsis (AUC) inset at right (*n*=4 lines/genotype), Student’s *t-*test. *P<0.05, **P<0.01, ***P<.001, ****P<0.0001.

Reduced adiposity, improved glucose homeostasis and reduced circulating lipids are characteristics of the pro-longevity intervention known as dietary restriction (DR), defined as reduced food intake without malnutrition, and linked an overall metabolic shift to FAO.^22^ Previously, DR-like phenotypes including improved insulin sensitivity and increased oxidative stress resistance have been observed in CS mouse models.^23^ We used indirect calorimetry to test the hypothesis that FAO was increased in CX mice despite *ad libitum* access to food. CX mice consumed more O_2_ (Figure 2a) and produced more CO_2_ (Figure 2b) than control littermates, but without significant differences in physical activity (Supplementary Figure S2a) or food intake (Supplementary Figure S2b). Importantly, a reduced respiratory exchange ratio (RER), indicative of preferential use of fat or protein relative to carbohydrate, was observed (Figure 2c, Supplementary Figure S2c), but only during the dark phase and despite normal intake of a nutritionally complete diet with 70% calories in the form of carbohydrates. Ketone bodies were appropriately elevated in both fasted control and CX mice, suggestive of an intact fasting response in CX mice without evidence of malabsorption (Supplementary Figure S2d).

Gene expression profiling of skeletal muscle from fasted CX relative to control mice revealed a significant increase in many FAO-related genes (Figure 2d). Consistently, soleus muscle from CX mice had a significantly elevated capacity to oxidize the radiolabelled 16C-saturated fatty acid palmitate relative to controls (Figure 2e). Interestingly, soleus from CSA KO animals with a much milder metabolic phenotype displayed a trend toward increased FAO capacity, whereas soleus from XPA KO mice with no reported metabolic phenotype did not. *Ex vivo* FAO capacity was also significantly increased in livers of CX mice relative to controls (Figure 2f).

Because FAO can be driven by substrate availability *in vivo*, we next asked if increased FAO capacity was an intrinsic property of CX cells. To this end, primary, low-passage dermal fibroblasts (mouse dermal fibroblasts (MDFs)) were isolated from tails of pre-weaning heterozygote WT and CX mice. We note that cells from CS patients and related rodent models do not show defects in cellular proliferation or onset of premature cellular senescence,^24^ nor did we observe this in CX cells.

Under standard serum-free Seahorse media conditions with 11 mM glucose, CX MDFs had a higher oxygen consumption rate (OCR) than control cells (Figure 2g), consistent with increased mitochondrial OXPHOS, and slightly but not significantly reduced extracellular acidification rate (ECAR) indicative of aerobic glycolysis (Supplementary Figure S2e). Both measures were normalized to total cellular protein content, a marker of cell viability, which was not different amongst genotypes (Supplementary Figure S2f); mitochondrial content was slightly but not significantly increased in CX MDFs relative to control cells (Supplementary Figure S2g). Under low-glucose conditions (2.5 mM), addition of the fatty acid palmitate increased OCR to a significantly higher level in CX MDFs compared with control cells (Figure 2h). Thus, increased OXPHOS activity appears to be a cell-intrinsic property of CX cells independent of the substrate, glucose or fatty acid that is available for oxidation.

### Increased FAO is a cell-autonomous, adaptive response triggered by genotoxic stress in CX cells

**Figure 3:**
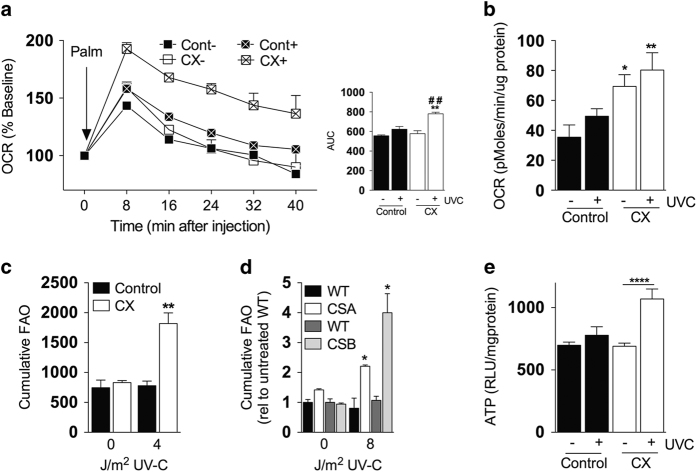
Increased fatty acid oxidation (FAO) is a cell-autonomous, adaptive response triggered by genotoxic stress in CX cells. (**a**) Oxygen consumption rate (OCR) over time after the injection of 200 μ mol/l bovine serum albumin (BSA)-conjugated palmitate in the indicated genotypes (*n*=3 independent mouse dermal fibroblasts (MDF) lines/genotype) 24 h post 4 J/m^2^ ultraviolet (UV)-C or mock treatment (+ or −, respectively) with AUC analysis at right; Student’s *t-*test between genotypes within UV treatment group**; Student’s *t-*test within genotype between ±UV treatment^##^. (**b**) OCR of MDFs (three lines/genotype) 24 h after 4 J/m^2^ UV-C or mock treatment; one-way analysis of variance (ANOVA) between genotypes within treatment group with Dunnett's post-test. (**c**) Cumulative FAO of tritiated palmitate over a 4 h period, 24 h after treatment of MDFs (*n*=3 lines/genotype) with indicated dose of UV-C; Student’s *t-*test between genotypes within UV dose. (**d**) Primary human CSA and CSB dermal fibroblasts treated as in (**c**); Student’s *t-*test between genotypes within UV dose. (**e**) Steady-state ATP levels of MDFs 24 h after exposure to 0 or 4 J/m^2^ UV-C; Student’s *t-*test within genotype between ±UV treatment. *P<0.05, **P<0.01, ****P<0.0001, ^##^*P*<0.01.

As increased FAO in CX cells/animals could be due to changes distinct from accumulated unrepaired DNA lesions, e.g., defects in transcriptional regulation associated with some forms of CS,^25^ we next tested the potential of DNA damage to directly impact OXPHOS/FAO capacity. To this end, MDFs were challenged with UV-C to induce bulky DNA lesions without generation of free radicals that can damage lipids and proteins. Twenty-four hours after a relatively low dose of 4 J/m^2^ UV-C versus mock treatment, OCR was significantly increased as a function of UV-C exposure in CX MDFs under conditions favoring FAO (2.5 mM glucose, addition of palmitate; Figure 3a, AUC inset) or glucose oxidation (11 mM glucose, without palmitate; Figure 3b), consistent with metabolic substrate flexibility as observed above. OCR readings were normalized to total protein content calculated from the same cells immediately after the final reading; protein content was not significantly altered by low-dose UV-C in this time frame (Supplementary Figure S3a). Mitochondrial content normalized to total protein was increased slightly but significantly on UV-C in both cell types (Supplementary Figure S3b), and thus unlikely to be causative of the specific increase in OCR upon UV-C in CX MDFs. ECAR was significantly reduced in UV-C treated CX cells compared with untreated WT cells (Supplementary Figure S3c).

Direct measurements of FAO capacity in dermal fibroblast cultures from CX mice (Figure 3c), as well as CSA and CSB primary human diploid fibroblasts (HDF; Figure 3d), revealed significant increases 24 h after UV-C irradiation. Increased FAO capacity was not specific to dermal fibroblasts, but was also observed on UV-C irradiation in CX-derived pre-adipocytes (Supplementary Figure S3d). Consistent with the potential for increased FAO/OXPHOS to impact energy homeostasis on genotoxic stress, steady-state ATP levels did not differ significantly amongst genotypes in unirradiated cells, but were significantly higher 24 h post irradiation in CX cells (Figure 3e), potentially reflecting increased energy production, decreased energy expenditure or both. Taken together, these data support a model in which increased FAO/OXPHOS is a cell-autonomous adaptation to both endogenous and exogenous genotoxic stress in CX cells with the potential to impact energy homeostasis.

### Increased FAO is a general response to acute genotoxic stress

**Figure 4:**
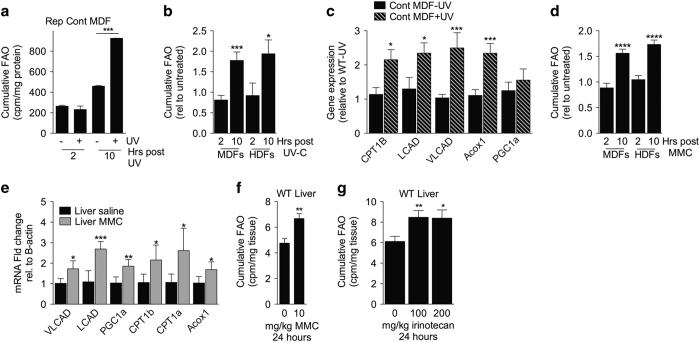
Increased fatty acid oxidation (FAO) is a general response to acute genotoxic stress. (**a**) Cumulative FAO of tritiated palmitate of a representative control mouse dermal fibroblast (MDF) line (Rep Cont MDF) over the indicated time period after exposure to 0 or 20 J/m^2^ UV-C; Student’s *t-*test between treatment groups within time point. (**b**) Cumulative FAO of tritiated palmitate of two additional UV-C treated control MDF lines expressed relative to untreated at the indicated time point post-irradiation, and of 3 UV-C treated WT human diploid fibroblast (HDF) lines relative to untreated at the indicated time; Student’s *t-*test. (**c**) messenger RNA (mRNA) expression of FAO-related genes in control MDFs 6 h after exposure to 20 J/m^2^ UV-C expressed relative to mock irradiated cells; Student’s *t-*test. (**d**) Cumulative FAO of tritiated palmitate of three independent MDF and HDF lines exposed to 40 μ mol/l mitomycin C (MMC) expressed relative to untreated at the indicated time point; Student’s *t-*test. (**e,f**) Gene expression of FAO-related genes (**e**) and FAO capacity (**f**) in liver from 8 week WT mice injected intraperitoneally (IP) with 10 mg/kg MMC or saline and harvested after 24 h (*n*=4/group); Student’s *t-*test between treatment groups. (**g**) FAO capacity of liver from 8 week WT mice injected IP with 100 or 200 mg/kg irinotecan or saline and harvested after 24 h (*n*=4/group); Student’s *t-*test. *P<0.05, **P<0.01, ***P<0.001, ****P<0.0001.

We next asked if the observed increase in FAO was a specific property of NER deficiency, or a general response to UV-C. As WT cells tolerate the low doses of UV-C used to characterize metabolic changes in CX cells, we exposed WT MDFs and HDFs to higher UV-C doses (20 and 24 J/m^2^, respectively) and measured the time-dependent increase in radiolabelled palmitate oxidation. Although no differences in the capacity to burn fatty acid were observed between irradiated and unirradiated MDFs within the first 2 h after UV-C exposure, by 10 h irradiated MDFs had oxidized significantly more fatty acid (Figure 4a) despite an overall time-dependent decrease in oxidation rates (Supplementary Figure S4a) likely due to unfavorable culturing conditions (e.g., low glucose, no serum) required for FAO measurements. Additional independent WT MDF and HDF cultures showed similar cumulative increases in FAO between 2 and 10 h after high-dose UV-C exposure (Figure 4b; Supplementary Figure S4b,c) coincident with an increase in expression of FAO-related genes 6 h after exposure in WT MDFs (Figure 4c).

Having established that increased FAO occurs upon high doses of UV-C irradiation of WT cells, we next asked if increased FAO is specific to helix-distorting UV lesions or a more general response to genotoxic stress. Treatment with the crosslinking agent MMC resulted in a similar increase in FAO in WT and CX MDFs (Supplementary Figure S4d) as well as in WT MDFs and HDFs (Figure 4d; Supplementary Figure S4e,f). Interestingly, IR, which induces mostly oxidative lesions and some double-stranded DNA breaks, failed to increase FAO capacity in MDFs at doses of 4.6 and 10 Gy, but significantly increased FAO capacity at 100 Gy (Supplementary Figure S4g,h). All cumulative measurements were normalized to cellular protein in the same well (Supplementary Figure S4i–k).

Finally, we asked if increased FAO is a physiological response to DNA damage *in vivo*, e.g., in the context of genotoxic chemotherapy. To this end, 9-week-old WT mice were treated with a single chemotherapeutic dose of MMC. One day later, MMC-treated mice displayed significantly increased FAO-related gene expression in liver and muscle (Figure 4e, Supplementary Figure S4l) and an increased FAO capacity in liver *ex vivo* (Figure 4f). Another chemotherapeutic, irinotecan (topoisomerase I inhibitor), also increased FAO capacity in liver and muscle 24 h after treatment (Figure 4g, Supplementary Figure S4m), suggesting increased FAO is a general response to genotoxic stress in primary cells *in vitro* and tissues *in vivo*.

### PARP-1-dependent NAD+/ATP depletion and AMPK activation link DNA damage to increased FAO

**Figure 5:**
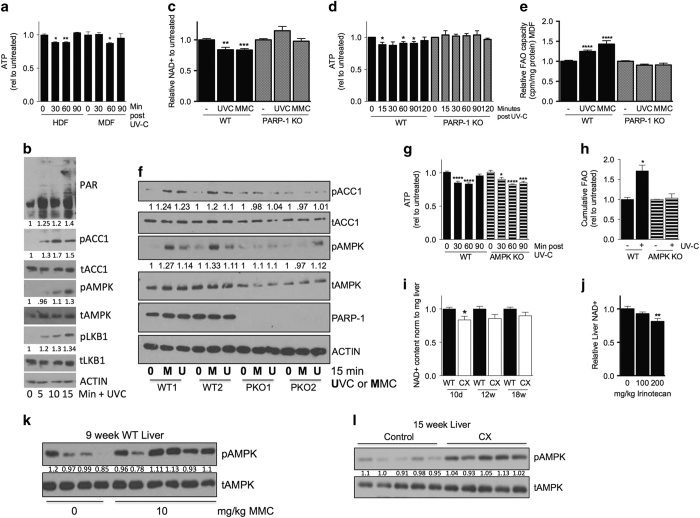
PARP-1-dependent NAD+/ATP depletion and AMPK activation link DNA damage to increased fatty acid oxidation (FAO). (**a**) Steady-state ATP levels of wild-type (WT) human diploid fibroblasts (HDFs) and mouse dermal fibroblasts (MDFs) after ultraviolet (UV)-C exposure (24 J/m^2^ for HDF and 20 J/m^2^ for MDFs); one-way analysis of variance (ANOVA) with Dunnett’s multiple comparisons test. (**b**) Western blot of WT MDFs collected 0, 5, 10 and 15 min after exposure to 20 J/m^2^ UV-C. (**c**) Relative NAD+ content measured in WT and PARP-1 KO MDFs (*n*=4–8 lines/genotype) 5 min after treatment with 20 J/m^2^ UV-C or 40 μ mol/l mitomycin C (MMC); Student’s *t-*test relative to untreated within genotype. (**d**) Steady-state ATP levels of WT and PARP-1 KO MDFs (*n*=3–4 lines/genotype) over a time course after exposure to 20 J/m^2^ UV-C; one-way ANOVA with Dunnett’s multiple comparisons test. (**e**) Relative cumulative FAO in WT and PARP-1 KO MDFs 10 h after exposure to 20 J/m^2^ UV-C or 40 μ mol/l MMC (*n*=3–4 lines/genotype); Student’s *t-*test relative to untreated within genotype. (**f**) Western blot of two independent WT and PARP-1 KO MDF lines collected 15 min after exposure to ±40 μ mol/l MMC or 20 J/m^2^ UV-C. (**g**) Steady-state ATP levels of WT and AMPKα1KO MDFs over a time course after exposure to 20 J/m^2^ UV-C (*n*=3–4 lines/genotype); one-way ANOVA with Dunnett’s multiple comparisons test. (**h**) Relative cumulative FAO in WT and AMPKα1KO MDFs 10 h after exposure to 20 J/m^2^ UV-C (*n*=3 lines/genotype); Student’s *t-*test relative to untreated within genotype. (**i**) Relative NAD+ content in livers from WT and CX mice (*n*=3–4/genotype) at the indicated ages; Student’s *t*-test between genotypes within age. (**j**) Relative NAD+ content in livers from WT mice intraperitoneally (IP) injected with 100 or 200 mg/kg irinotecan or saline and harvested after 24 h (*n*=4/group); Student’s *t-*test relative to untreated. (**k**) Western blot of livers from 8 week WT mice injected IP with MMC or saline and harvested after 24 h (*n*=4/group);. (**l**) Western blot of 15 week WT and CX livers. *P<0.05, **P<0.01, ***P<0.001, ****P<0.0001.

How does DNA damage trigger a metabolic switch to increased FAO/OXPHOS? Because FAO provides reducing equivalents for efficient ATP generation via mitochondrial OXPHOS, we hypothesized that the rapid increase in FAO/OXPHOS was an adaptive response to increased energy demands on genotoxic stress. Consistent with this, steady-state ATP levels were transiently reduced in WT MDFs and HDFs within 30–60 min of UV-C or MMC treatment, returning to initial levels within 60–90 min (Figure 5a, Supplementary Figure S5a).

Multiple genotoxins result in activation of PARP-1,^13^ which consumes NAD+ to synthesize PAR chains on target proteins surrounding DNA breaks. The resulting NAD+ depletion can mediate ATP loss through multiple mechanisms, including inhibition of ATP generation by glycolysis for which NAD+ is a key cofactor.^12^ To test the potential of PARP-1 activation to contribute to ATP depletion, we first confirmed its activity by measuring PAR accumulation and NAD+ depletion in WT versus PARP-1 KO primary dermal fibroblasts subject to genotoxic stress. Increased PARylation was observed in the nucleus by immunofluorescence (Supplementary Figure S5b) and in whole cell extracts by western blot (Figure 5b, Supplementary Figure S5c,d) within minutes of MMC or UV-C treatment and coincident with a significant reduction in NAD+ levels (Figure 5c) in WT but not PARP-1 KO cells.

Importantly, transient ATP depletion and increased FAO capacity observed upon two different forms of genotoxic stress in WT cells were both absent in PARP-1 KO cells (Figure 5d,e, Supplementary Figure S5e,f). These data suggest that PARP-1 may serve as the molecular link between DNA damage and energy metabolism across a spectrum of DNA lesions, likely by binding to a DNA damage repair intermediate common to these different lesions.

AMPK controls metabolic adaptations to ATP depletion including decreased energy expenditure, altered substrate utilization and increased energy efficiency production via OXPHOS.^15^ Consistent with a role for AMPK in metabolic reprogramming in response to DNA damage, MDFs subject to genotoxic stress displayed a rapid, time-dependent increase in phosphorylated/activated AMPK (Figure 5b) in a PARP-1-dependent manner (Figure 5f, Supplementary Figure S5d).

To test the requirement for AMPK in metabolic reprogramming on genotoxic stress, we used primary dermal fibroblasts isolated from WT and AMPKα1KO mice which lacked detectable expression of the AMPK catalytic subunit (Supplementary Figure S5g). Steady-state ATP levels were reduced in both WT and AMPKα1 KO MDFs in response to 20 J/m^2^ UV-C or 40 μM MMC, but returned to baseline within 2 h only in WT cells (Figure 5g, Supplementary Figure S5h), consistent with the inability of AMPKα1 KO cells to maintain energy homeostasis. AMPKα1 KO MDFs also failed to activate FAO within 10 h of various genotoxin treatments as in WT cells (Figure 5h, Supplementary Figure S5i,j) without consistent effects on cell viability (Supplementary Figure S5k–m).

To test the potential relevance of PARP-1-mediated NAD+ depletion and AMPK activation in metabolic reprogramming in response to acute or chronic DNA damage *in vivo,* we analyzed livers from CX versus WT mice at different ages, and from WT mice 24 h after treatment with a chemotherapeutic genotoxin, irinotecan or MMC. In all cases, we observed reduced steady-state NAD+ levels (Figure 5i,j) and increased phospho-AMPK (Figure 5k,l).

### Increased FAO is a beneficial adaptive response to genotoxic stress

**Figure 6:**
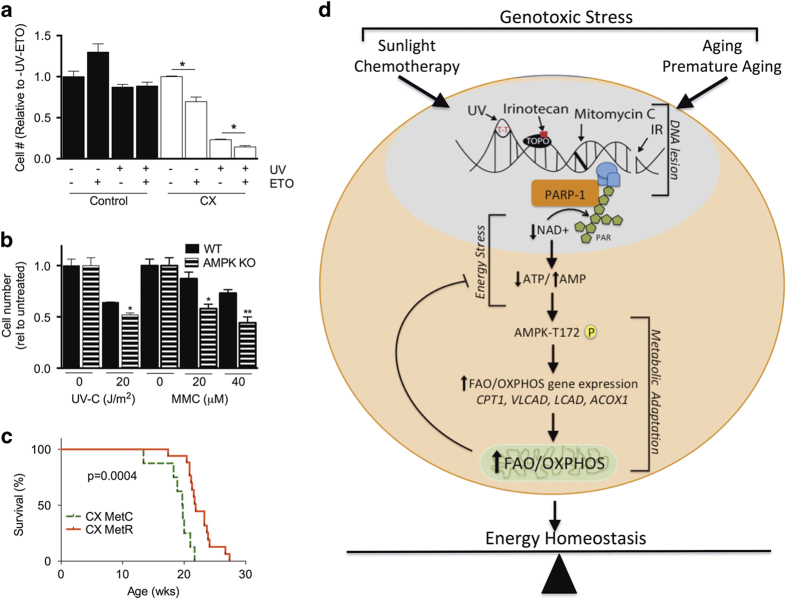
Increased fatty acid oxidation (FAO) is a beneficial adaptive response to genotoxic stress. (**a**) Relative MDF cell number 24 h after 20 Jm/^2^ UV-C and/or 40 μM Etomoxir treatment (*n*=2 independent MDF lines/genotype in duplicate); Student’s *t-*test between indicated groups. (**b**) Relative cell number of WT and AMPKα1KO MDFs 24 h after ultraviolet (UV)-C or mitomycin C (MMC; *n*=2 MDF lines/genotype in duplicate); Student’s *t-*test versus untreated within genotype and treatment. *P<0.05, **P<0.01. (**c**) Kaplan–Meier survival plots of CX mice on methionine restricted (MetR; *n*=17) or methionine complete (MetC; *n*=8) diets; log-rank test *P*=0.0004. (**d**) Model of relationship between DNA damage and maintenance of cellular and organismal energy homeostasis. Acute and chronic genotoxic stress activate PARP-1, depleting steady-state ATP and NAD+ levels, activating AMPK and increasing FAO/OXPHOS to restore energy homeostasis.

To determine if increased FAO is a beneficial or maladaptive response to genotoxic stress, we determined cellular hypersensitivity to genotoxic stress *in vitro* in the presence or absence of the FAO inhibitor, etomoxir. In addition to hypersensitivity to 20 J/m^2^ UV-C, CX MDFs displayed reduced viability upon etomoxir treatment in both mock and UV-C treated groups (Figure 6a), consistent with beneficial effects of increased FAO upon both chronic and acute genotoxic stress. A similar hypersensitivity to UV-C and MMC was observed in AMPKα1KO MDFs relative to WT cells (Figure 6b) at doses that increased FAO in WT cells.

Because blocking whole-body FAO *in vivo* is technically challenging and potentially toxic, we probed the *in vivo* role of FAO by testing the hypothesis that increased FAO is a beneficial adaptive response to chronic DNA damage in CX mice. To this end, we employed a diet limited for the essential amino acid methionine, which, like DR, promotes a metabolic shift to FAO.^26^ After 2 weeks of methionine restriction (MetR), RER was decreased in CX relative to control mice during the dark phase (Supplementary Figure S6a) without affecting activity (Supplementary Figure S6b) or food intake (Supplementary Figure S6c), consistent with an increase in FAO. Separate cohorts of CX and WT mice on MetR diets from ~6 weeks also displayed a reduction of body weight (Supplementary Figure S6d) and specifically of the percentage fat mass (Supplementary Figure S6e), with a reciprocal increased percentage lean mass (Supplementary Figure S6f) relative to within genotype controls on the control (MetC) diet. Absolute fat and lean mass also displayed similar trends between genotypes (Supplementary Figure S6g,h). Despite total body fat at or below the level of detection by EchoMRI in the MetR CX group from ~15 weeks of age, MetR significantly increased median and maximum lifespan of CX mice by ~2 and 5 weeks, respectively (mean 19.8 weeks MetC versus 21.9 weeks MetR; maximum 21.7 weeks MetC versus 27.4 weeks MetR; log-rank test *P*=0.0004; Figure 6c). Although the effects of MetR are pleiotropic and extend beyond increased FAO, we can clearly conclude that neither increased FAO nor loss of adiposity exacerbate CX symptoms *in vivo*; on the contrary, the pro-longevity effects of MetR in this short-lived DNA repair-deficient model are consistent with increased FAO as a beneficial, adaptive response to chronic genotoxic stress *in vivo*. Taken together, these data support a model in which an AMPK-dependent increase in FAO/OXPHOS is an adaptive response to DNA damage-mediated PARP-1 activation that promotes maintenance of NAD+/ATP energy homeostasis and cell survival (Figure 6d).

## Discussion

### Increased FAO/OXPHOS is a cell-autonomous response to DNA damage

Here, we identified a novel link between DNA damage and adaptive cellular/organismal energy metabolism involving an increase in FAO in particular and OXPHOS in general. This connection was observed on acute treatment of WT animals with a variety of genotoxins giving rise to distinct spectrums of DNA lesions, as well as in a model of chronic genotoxic stress induced by congenital DNA repair deficiency. It was also observed upon treatment of different types of primary cells, both human and mouse, with these different genotoxins. Based on these considerations, we conclude that this metabolic response represents a general, cell-autonomous response to acute and chronic genotoxic stress.

Why do cells increase OXPHOS on DNA damage, and what is the molecular mechanism linking DNA damage to altered energy metabolism? Our data suggest that multiple different DNA lesions (or potentially common repair intermediates) activate PARP-1, and that PARP-1-dependent activation of AMPK serves as the link between DNA damage and adaptive changes in NAD+ and ATP energy metabolism. Although PARP-1 activation is directly linked to NAD+ depletion, the definitive cause of ATP loss upon PARP-1 activation remains elusive. Several non-mutually exclusive possibilities have been described. NAD+ biosynthesis, either *de novo* or via the salvage pathway, is an ATP-dependent process, although its contribution to ATP depletion has recently been contested.^12^ Free PAR that is rapidly removed from PARylated proteins can bind to and inhibit hexokinase-1, thus blocking ATP production via glycolysis.^12^ Furthermore, free AMP released upon PAR degradation, e.g., by PARG, could compete with ADP for binding to the adenine nucleotide transporter and further diminish ATP production; consistent with such a possibility, methylnitronitrosoguanidine (MNNG)-induced PAR accumulation and PARG activation results in a time-dependent increase in AMP concentrations and a concomitant decrease in ATP in HeLa cells.^27^ Regardless of the mechanism, each of these could affect ATP levels and thus promote AMPK activation, which contributes to energy homeostasis by blocking anabolic ATP-consuming processes and increasing efficient energy production via mitochondrial OXPHOS, including increased FAO. Although AMPK was previously shown to be activated by phosphorylation on DNA damage,^13^ this is the first demonstration of a role for energy stress itself in DNA damage-related AMPK activation.

It is important to note that while we observed an increase in FAO/OXPHOS as a common response to multiple genotoxic stressors in a variety of primary cells *in vitro*, as well as in models of acute and chronic DNA damage *in vivo*, this response was not uniform across doses of all agents. For example, although 100 Gy IR activated FAO, 5–10 Gy failed to activate FAO despite inducing considerable DNA damage and activating cellular senescence.^28^ This could be explained in part by the previous observation that PARP-1 activation on IR only occurs above 20 Gy.^6^ Furthermore, low-dose IR (<0.1 Gy) activates a HIF1α-dependent beneficial adaptive response involving increased glucose uptake and glycolysis *in vivo*.^29^ DNA damage can also block mitochondrial glutamine anaplerosis via a SIRT4-dependent mechanism, limiting cell proliferation, although whether this has any effect on the balance between oxidative and glycolytic energy metabolism is not known.^30^ Future experiments will be required to determine if and how other forms of DNA damage or DNA repair deficiencies perturb energy metabolism.

### Increased FAO/OXPHOS is a beneficial adaptive response to DNA damage

Changes in FAO can be driven either by metabolic inflexibility, e.g., on insulin resistance^31^ or other constitutive defects in glucose metabolic regulation,^32^ or as an adaptive response to environmental cues, ranging from differential substrate availability in response to normal feeding/fasting cycles^33^ or overall restricted food intake.^34^ Our data in the CX model, including improved HOMA-IR *in vivo* and increased oxygen consumption in cells independent of energy substrate *in vitro,* are inconsistent with metabolic inflexibility. However, two observations made in this study strongly suggest that increased FAO/OXPHOS is a beneficial adaptive response to genotoxic stress. First, blocking AMPK or FAO reduced cell viability on genotoxic stress; second, increasing FAO extended lifespan in a short-lived mouse model of chronic genotoxic stress.

Interestingly, previous studies on XPA as well as CX mice point to PARP-1 hyperactivation and NAD+ depletion driving mitochondrial dysfunction subsequent to defective SIRT1-dependent mitophagy.^35^ Similalry, age-related accumulation of damaged mitochondria has been observed in the CSB mouse model.^36^ Although these findings may appear at odds with the beneficial metabolic adaptations involving overlapping molecular pathways as described here, they are not necessarily mutually exclusive. We suggest that adaptive changes in energy metabolism may precede constitutive defects in mitochondrial function, but are insufficient to prevent such defects because they fail to address the fundamental defect in DNA repair. Future studies will be required to address this and other possibilities. Nonetheless, our findings have important translational implications for conditions driven by chronic or acute DNA damage as discussed below.

For CS and related progeroid syndromes, these results have implications for our understanding of pleiotropic disease symptoms as well as potential treatment options. Loss of subcutaneous fat is often observed in segmental progeroid disorders such as CS, and has been generally interpreted as a maladaptive response to the rapid aging process. Reduced adiposity and increased FAO in CX mice is unlikely due to any underlying defect in glucose homeostasis, as the response to glucose challenge was normal, and HOMA-IR improved, in CX mice. Instead, our data suggest that loss of adiposity is a constituent of the beneficial adaptive response driven in part by increased fat burning. Consistent with this interpretation, the MetR diet significantly extended longevity of short-lived CX mice despite a further reduction of adiposity. Interestingly, HFD feeding rescues age-dependent hearing loss in a CS model with mild symptoms relative to CX mice.^37^ At face value, beneficial results from varying dietary interventions, HFD and MetR, appears paradoxical. However, under the working assumption that DNA damage-driven energy stress is a major contributor to both hearing loss and reduced lifespan, it then follows that improved maintenance of energy homeostasis either by energy supplementation (HFD) or increased energy efficiency (MetR) could both have beneficial effects. Future experiments will be required to determine if this metabolic adaption to genotoxic stress is present in CS patients and potentially other diseases and their corresponding mouse models with defects in genome maintenance.^38^ However, existing data collectively suggest that strategies to improve energy homeostasis, either by pharmacologic (NAD+ supplementation) or dietary (DR, MetR, ketogenic diet) means, represent exciting new avenues toward mitigation of disease symptoms.

Finally, our findings here have implications for interventional approaches to protect against clinically relevant stressors involving genotoxic stress, ranging from radio/chemotherapeutics to oxidative stress associated with ischemia reperfusion injury. Dietary preconditioning is one such prophylactic approach involving short-term DR or fasting before chemotherapy or surgical stress that significantly reduces detrimental side effects of these interventions;^39–41^ however, the mechanisms of protection remain unclear. Our data suggest the possibility that activation of FAO/OXPHOS by diet before genotoxic stress may contribute to protection by preserving energy homeostasis and cell viability, and further that this mechanism may function more effectively when activated before the onset of DNA damage.

## Materials and methods

### Mice

All mouse experiments were performed with the approval of the Harvard Medical Area Institutional Animal Care and Use Committee. Mice carrying *Xpa* and/or *Csa* knockout alleles in a C57BL/6 background, AMPKα1 (Jackson 014141 bred with E2a-cre) knockout mice in a C57BL/6 background and PARP-1 knockout mice (Jackson 002779) on a 129S background were maintained under standard laboratory conditions (temperature 20–24 °C, relative humidity 50–60%, 12 h light/12 h dark) and allowed free access to food (Research Diets D12450B with 10% calories from fat, 18% from casein and 72% from carbohydrate) and water unless otherwise indicated as described previously.^19^ Other diets utilized include facility chow pellets (PicoLab 5058, Purina), HFD (research diets, 60% calories from fat), and a 1% agar-based diet using D12450BSpx (research diets) and individual crystalline amino acids (Ajinomoto) in proportions to those in casein. MetR diets contained 1.5 g Met/kg food and lacking Cys (ref. 42) compared with MetC diet with the same amino acid composition except for 4.5 g Met/kg food. MMC and irinotecan treatment of WT mice was performed by intraperitoneal (IP) injection with 10 mg/kg MMC or 100/200 mg/kg irinotecan in saline.

### Cell lines

MDFs were passaged in 20% fetal bovine serum (FBS) in DMEM+Penicillin/Streptomycin (P/S). Primary human dermal fibroblasts (HDFs) from CS-B patients were a gift from PJ Brooks (CSB: GM00739C and WT: RB4492); CS-A HDFs were purchased from Coriell Cell Repository (CSA: AG10213 and WT: AG10215, AG12726; CSB: AG12724) and passaged in 15% FBS DMEM+P/S. All cell lines were incubated in 5% CO_2_ and 3% O_2_. MDFs were treated with 4 or 20 J/m^2^ UV-C and HDFs at 8 or 24 J/m^2^; both were treated with 4.6, 10 or 100 Gy IR, 40 μ mol/l MMC, 40 μ mol/l compound C and 40 μ mol/l Etomoxir (Sigma, St Louis, MO, USA). Cell survival was measured by the relative change in cell number as measured on a hemocytometer following plating of an equal number of cells per line.

### Seahorse

Cellular OCR and ECAR were measured using the Seahorse Cell Metabolism Analyzer XF96. Cells were untreated or irradiated as described above and plated at a density of 18,000 cells/well in a 96-well plate. After 24 h, media changed to unbuffered XF assay media with 11 m mol/l glucose, 2 m mol/l glutamine and pyruvate at pH7.4 and basal OCR and ECAR measured for blocks of 2 min mixing and 5 min measuring. For palmitate assays, cells were treated ±UV-C, plated at 18,000 cells/96 well and 24 h later media changed to KHB buffer (0.5 m mol/l KCl, 11.1 m mol/l NaCl, 0.2 m mol/l MgSO_4_, 0.14 m mol/l NaH_2_PO_4_ and 500 μ mol/l Carnitine pH7.4) with 2.5 m mol/l glucose. After three readings at 2 min mixing and 5 min measuring, either bovine serum albumin (BSA) or BSA-conjugated palmitate was injected at 200 μ mol/l final concentration, and five more readings recorded. All plates were normalized to protein content as measured after Seahorse by bicinchoninic acid assay (BCA).

### Fatty acid oxidation in tissues

Soleus and liver were removed, weighed and placed in KH buffer containing 25 m mol/l NaHCO_3_, 118 m mol/l NaCl, 4.7 m mol/l KCl, 1.2 m mol/l MgSO_4_, 1.2 m mol/l NaH_2_PO_4_, 1.2 m mol/l CaCl_2_ and glucose (2.5 m mol/l). The tissues were kept in buffer on ice until all dissections were completed. Each tissue then transferred to KH buffer plus 2% fatty-acid-free BSA and 2.5 m mol/l glucose, with 2 μCi ^3^H palmitic acid and incubated at 37 °C for 1 h. The buffer was collected and hydrolyzed ^3^H palmitic acid (as ^3^H water) was extracted. Buffer (100 μl ) was added to 100 μl of 10% trichloroacetic acid, vortexed, incubated at RT for 15 min, spun at 16,000 r.p.m. for 10 min, and the supernatant collected into a new tube. Trichloroacetic acid (5%; 100 μl) and 40 μl BSA (10%) was added to the supernatant, vortexed, incubated at RT for 15 min, spun at 16,000 rpm for 10 min, and the supernatant transferred to a new tube. Chloroform:methanol (2:1, 750 μl) was added to the supernatant, along with KCl: HCl (2 mol/l each, 300 μl), vortexed and spun at 16,000 r.p.m. for 10 min. The upper layer (~600 μl) was collected into 5 ml EcoLume, mixed, and counted in a liquid scintillation counter. After subtracting background cpm, the sample cpm was divided by the tissue weight to determine FAO capacity.

### Body composition and metabolic parameters

An EchoMRI device (Echo Medical System, Houston, TX, USA) was used to determine lean and fat mass. Insulin tolerance tests were performed after a 4–5 h fast by injection of 0.5 units insulin/kg body weight into the peritoneum. Glucose tolerance tests were performed after 4–6 h fast by injection of 2 mg/kg glucose IP. Blood glucose was measured before and after from tail blood at indicated time points using the OneTouch glucose monitoring system (Lifescan, Milpitas, CA, USA). Serum insulin (Alpco, Salem, NH, USA), leptin (R&D Systems, Minneapolis, MN, USA) and adiponectin (R&D Systems) were measured by enzyme-linked immunosorbent assay. Serum triglycerides (Sigma) and beta-hydroxybutyrate (Pointe Scientific, Canton, MI, USA) were measured by enzymatic kits. HOMA-IR was determined using the mouse serum insulin ELISA kit (Alpco).

### MDF isolation

MDFs from pre-weaning WT, CSA, XPA, CX and PARP-1 knockout mice and 20wk WT and AMPKα1KO mice were isolated as described previously.^24^ In brief, tail skin was removed and minced using a razor blade in the presence of 1.6 mg/ml collagenase II in 20% FBS DMEM P/S and incubated overnight in one well of a 6-well plate at 37 °C in a 5% CO_2_ and 3% O_2_ incubator. After ~16 h, the minced tissue was pipetted up and down 50 times, passed through a 70 μm filter, spun down and resuspended in the above media without collagenase. After a day or two, cells were trypsinized and replated in 2 wells of a 6-well plate. Cells were expanded after culture to 90% confluency and utilized between passages 3 and 5.

### Indirect calorimetry

Animals were placed in metabolic cages (Comprehensive Lab Animal Monitoring System (CLAMS), Columbus Instruments, Columbus, OH, USA) for 2 days and data collected from the beginning of the first light cycle. Oxygen consumption and carbon dioxide production were normalized to total lean mouse weight. The RER was determined by the ratio of CO_2_ produced (VCO_2_) over O_2_ consumed (VO_2_), both corrected for lean mass. RER values were averaged during the light and dark cycles on a per animal basis. Food consumed was corrected for lean mass of mouse over the 2 day period.

### Pre-adipocyte isolation and FACS

Subcutaneous fat pads from WT, CSA, XPA and CX mice at 14–16 weeks of age were isolated, washed in phosphate-buffered saline (PBS) and minced. Tissue was digested for 60 min with 1 mg/ml collagenase II in Hanks' balanced salt solution with 7.5% FBS then passed through a 70 μm filter and spun at 400 *g* for 10 min. The floating adipocytes were removed and the pelleted stromal vascular fraction was resuspended in erythrocyte lysis buffer (154 m mol/l NH_4_Cl, 5.7 m mol/l K_2_HPO_4_, 0.1 m mol/l EDTA) for 10 min. The cells were then pelleted again, washed in PBS and resuspended in 1:1 DMEM:Ham’s F12+P/S and 10% FBS. Media was changed every 2–3 days until 90% confluency was reached in ~7 days. For FACS analysis, erythrocytes were removed from stromal vascular fraction and 1 million cells were blocked with CD16/32 monoclonal antibody and stained for 30 min at 4 °C in the dark with F4/80 FITC and CD11b PeCy7 (at 1:100) all from BioLegend (San Diego, CA, USA). Cells were washed and acquired immediately on a BD FACSCalibur (BD Biosciences, San Jose, CA, USA) and analyzed with FlowJo (Tree Star, Ashland, OR, USA).

### Cellular fatty acid oxidation

Cells were plated at 100,000 cells/well in a 12-well plate in DMEM+P/S with 20% FBS for MDFs and 15% for HDFs. For low-dose (4–8 J/m^2^) treatments, cells were exposed to UV-C and then returned to standard media for 24 h. For high-dose UV-C (20–24 J/m^2^) and IR (4.6–100 Gy) treatments, cells were either exposed, washed with PBS, then incubated for 30 min with DMEM 2% fatty acid-free BSA and 5.5 m mol/l glucose or immediately given DMEM 2% fatty acid-free BSA, and 5.5 m mol/l glucose plus 40 μ mol/l MMC for 30 min. Media was then changed to DMEM 2% fatty acid-free BSA, 5.5 m mol/l glucose (±genotoxin), and 2 μCi ^3^H palmitic acid and incubated for 2–10 h. The media was then collected and hydrolyzed ^3^H palmitic acid (as ^3^H water) was extracted by the same technique as in tissues. After the media was removed, the cells were washed with 1× PBS and incubated for 10 min with 0.1 M NaOH at room temperature, and total protein collected for quantitation using BSA standards. After subtracting the background cpm, the sample cpm was divided by the protein concentration for a measure of cumulative FAO.

### ATP measurements

MDFs and HDFs were plated at a density of 10,000 cells per 96 well. Sixteen hours later, cells were dosed with UV-C, IR or 40 μ mol/l MMC. Up to 2 h later, 100 μl fresh media was added and ATP measured on the addition of 100 μl Cell Titer Glo reagent (Promega, Madison, WI, USA). The plate was shaken for 2 min and then incubated at room temperature in the dark for 10 min before determining the luminescence in a plate reader (Biotek Synergy 2). ATP content was normalized to protein content as determined by BCA.

### Mitochondrial content

18,000 MDFs were plated after 0 or 4 J/m^2^ UV-C treatment and placed at 37 °C in a 5% CO_2_, 3% O_2_ incubator for 24 h. Media was then changed to 80 nM Mitoview Green in standard MDF media for 30 min. Cells were washed with PBS and read on a plate reader.

### Quantitative real-time PCR

Total RNA was isolated from cells and tissues using Qiazol (Qiagen, Hilden, Germany) and complementary DNA synthesized by random hexamer priming with the Verso cDNA kit (Thermo Fisher, Waltham, MA, USA). quantitative real-time PCR was performed using Taq-Pro DNA polymerase (Denville Scientific, Holliston, MA, USA) and SYBR green dye (Lonza, Portsmouth, NH, USA). Fold changes were calculated by the ΔΔC_t_ method using B-actin as a standard, and normalized to the experimental WT control. Primer sequences are as follows: B-actin F: 5ʹ-AGCTTCTTTGCAGCTCCTTCGTTG R: TTCTGACCCATTCCCACCATCACA-3ʹ; CD36; F: 5ʹ-GAGCAACTGGTGGATGGTTT R: GCAGAATCAAGGGAGAGCAC-3ʹ; CPT1β F: 5ʹ-TTGCCCTACAGCTGGCTCATTTCC R: GCACCCAGATGATTGGGATACTGT-3ʹ; LCAD F:5ʹ-TCTTTTCCTCGGAGCATGACA R: GACCTCTCTACTCACTTCTCCAG-3ʹ; VLCAD F:5ʹ-CTACTGTGCTTCAGGGACACC R: CAAAGGACTTCGATTCTGCCC-3ʹ; PGC1α F: 5ʹ-AGCCGTGACCACTGACAACGAG R: GCTGCATGGTTCTGAGTGCTAAG-3ʹ; Leptin F: 5ʹ-TGAAGCCCAGGAATGAAGTC R: TCAAGACCATTGTCACCAGG-3ʹ; PPARα F: 5ʹ-TGTTTGTGGCTGCTATAATTTGC; R: GCAACTTCTCAATGTAGCCTATGTTT-3ʹ; Acox1 F: 5ʹ-CCTGATTCAGCAAGGTAGGG R: TCGCAGACCCTGAAGAAATC-3ʹ.

### Histology

Perigonadal white adipose tissue (WAT) depots were isolated, washed in PBS and fixed in Bouin’s solution for 24 h. Paraffin-embedded tissue was then sectioned and stained with hematoxylin and eosin for overall morphology or immunostained as previously described using rat anti-mouse F4/80 diluted 1:200 (eBioscience, San Diego, CA, USA). Controls were performed simultaneously.

### Westerns

Tissues and cells were homogenized with NP-40 buffer containing protease and phosphatase inhibitors and dithiothreitol (DTT). Samples were normalized for protein content, boiled with sodium dodecyl sulfate loading buffer separated by sodium dodecyl sulfate-polyacrylamide gel electrophoresis, transferred to polyvinylidene difluoride membrane (Whatman, Maidstone, UK) and blotted for phospho-ACC1 (S79, #3661), phospho-AMPK (T172, #2535), ACC1 (#4190), AMPK (#2532), phospho-LKB1 (#3482), LKB1 (#3050), Parp-1 (#9542) and Actin (#4967) from Cell Signaling Technologies (Danvers, MA, USA) and PAR (51–811HKC) from BD Biosciences (San Jose, CA, USA).

### Lipid profiling

Sera lipidomics profiling was conducted using liquid chromatography separations coupled with mass spectrometry based analysis on a Thermo Exactive Mass Spectrometer. Detailed methods have been described.^43,44^

### NAD+ determination

MDFs were plated at 500,000 per 10 cm plate. The following day media was removed, cells treated ±20 J/m^2^ UV-C, and then media returned. Five minutes later, cells were washed with PBS, trypsinized and NAD+ determined using the EnzyChrom NAD+/NADH Assay kit (E2ND-100, Bio-Assay systems). For tissues, approximately 20mg size pieces were prepared and used in the same assay and normalized to protein content by BCA.

### Immunofluorescence

MDFs were plated on 18 mm circular coverslips at 350,000 cells per well in a 12-well plate. Cells were treated with 40 μ mol/l MMC or 20 J/m^2^ UV-C, incubated for the indicated amount of time, rinsed twice with PBS and permeabilized 5 min at room temperature with 0.5% Triton in PBS. Cells were then fixed in 3.7% formaldehyde in PBS for 10 min and washed twice in PBS. Cells were then incubated for 10 min in 0.5% NP-40 in PBS, washed in PBS, then blocked in PBS+0.5% BSA (PBB) for 20 min. Primary antibody (1:200, anti-PAR clone 10H, Enzo Life Sciences, Farmingdale, NY, USA) was incubated in PBB overnight at 4 °C. Cells were then washed 3×5 min in PBB, incubated 1 h at 37 °C in secondary (goat anti-mouse 1:1000 Alexa Fluor Thermo Fisher) in PBB. Cells were washed 3×5 min in PBB, rinsed in PBS, and mounted with 4ʹ,6-diamidino-2-phenylindole on slides. Cells were imaged using a Zeiss LSM 700 confocal microscope, acquired using Zen Black software and analyzed using Fiji software.

### Statistics

The indicated statistical analyses were performed either in Excel or in GraphPad Prism.
