# Supplementary material for: Increased oxidative phosphorylation in response to acute and chronic DNA damage
Source: NPJ Aging Mech Dis. 2016 Oct 13;2:16022–. doi: 10.1038/npjamd.2016.22 (PMC5514997; doi:10.1038/npjamd.2016.22)
Supplement: Supplementary Figures [file npjamd201622-s1.pdf]

Supplemental Figure Legends

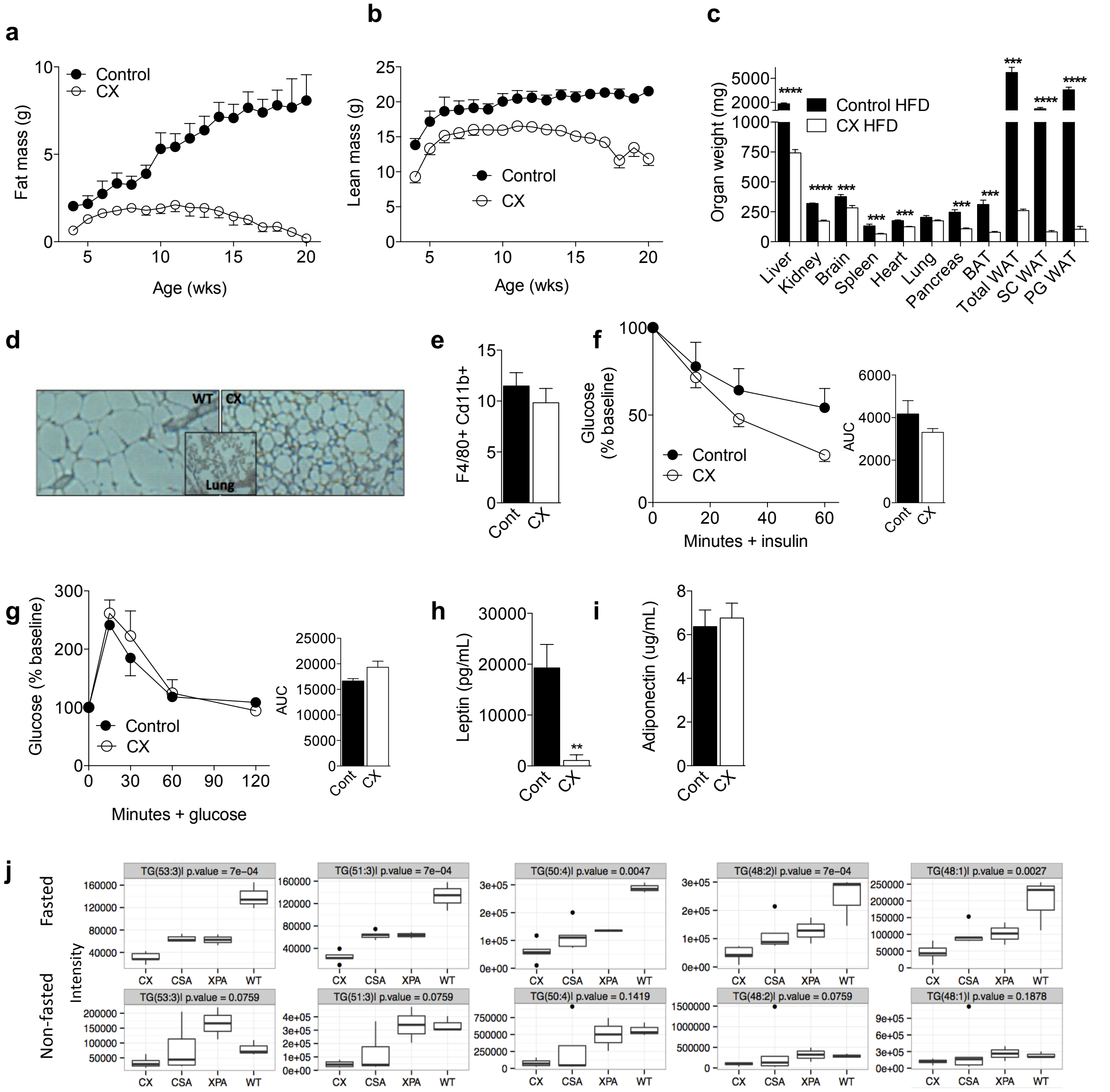

S1: Perturbations in energy metabolism indicative of improved metabolic fitness in CX mice. Absolute fat **(a)** and lean **(b)** mass of animals described in Figure 1a-c, n=14-16/group. **c.** Organ weights as described in Figure 1d expressed in mg absolute weight, n=4/group; Student's T test. **d.** F4/80 staining of paraffin-embedded white adipose tissue from Control (WT) and CX mice. Inset: lung tissue with F4/80 positive resident macrophages as a positive control. **e.** FACS analysis of F4/80+CD11b+ macrophages expresses as a percent of total cells in stromal vascular fraction from subcutaneous WAT from Control and CX mice as indicated, n=5-6/genotype, Student's T test. Insulin tolerance test **(f)** and glucose tolerance test **(g)** of 14wk Control and CX mice (n=3-4/group). Right: Area under the curve (AUC) of the indicated groups. **h.** Serum leptin levels in 12-16wk old Control and CX mice n=4/group; Student's T test. **i.** Serum adiponectin levels in 12-16wk old Control and CX mice n=5/group. **j.** Relative intensity of fed and fasted serum triglycerides as described in Figure 1i separated by genotype; 2-way ANOVA with Bonferroni post-test. **p<0.01, ***p<0.001, ****p<0.0001.

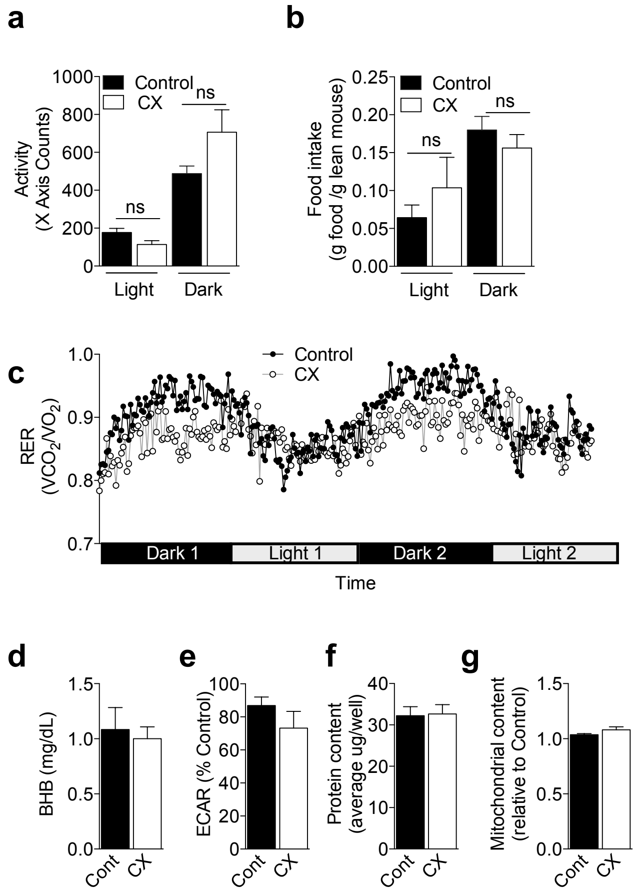

S2: Increased fatty acid oxidation in CX mice *in vivo* and in cells *in vitro*. Horizontal activity **(a)** and food consumption **(b)** in metabolic cages as described in Figure 2a-c, n=12/group. **c.** Respiratory exchange ratio in metabolic cages over 2 days in a representative experiment with 4 animals per genotype as described in Figure 2c. **d.** Concentration of beta-hydroxybutyrate in fasted serum from 12-16wk old control and CX mice, n=12/group. **e.** Extracellular acidification rate (ECAR) expressed as a percentage of Control MDFs from Figure 2g, n=3/genotype. **f.** Protein content by BCA of cells described in Figure 2g after Seahorse analysis, n=3/genotype. **g.** Relative mitochondrial content by Mitoview Green of cells described in Figure 2g, n=3/genotype.

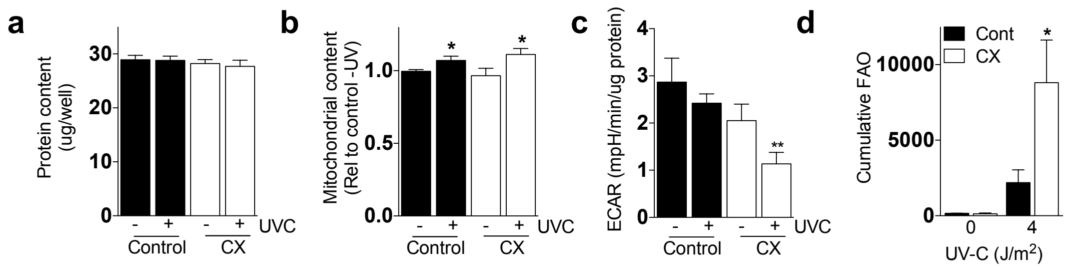

S3: Increased FAO is a cell-autonomous beneficial, adaptive response triggered by genotoxic stress in CX cells. a. Protein content of cells described in Figure 3a immediately after Seahorse analysis, n=3 independent MDF lines/genotype. **b.** Relative mitochondrial content 24hrs after 0 or 4J/m^2 UV-C; Student's T test within genotype between treatment groups, n=5/group. **c.** Extracellular acidification rate (ECAR) of cells described in Figure 3b expressed as a percentage of Control untreated cells; 1way ANOVA between genotypes within treatment group with Dunnett's post test, n=3 lines/genotype. **d.** Cultured pre-adipocytes from subcutaneous WAT SVF of control and CX mice and treated as in 3c; Student's T test within genotype between +/- UV treatment, n=3 lines/genotype. *p<0.05, **p<0.01.

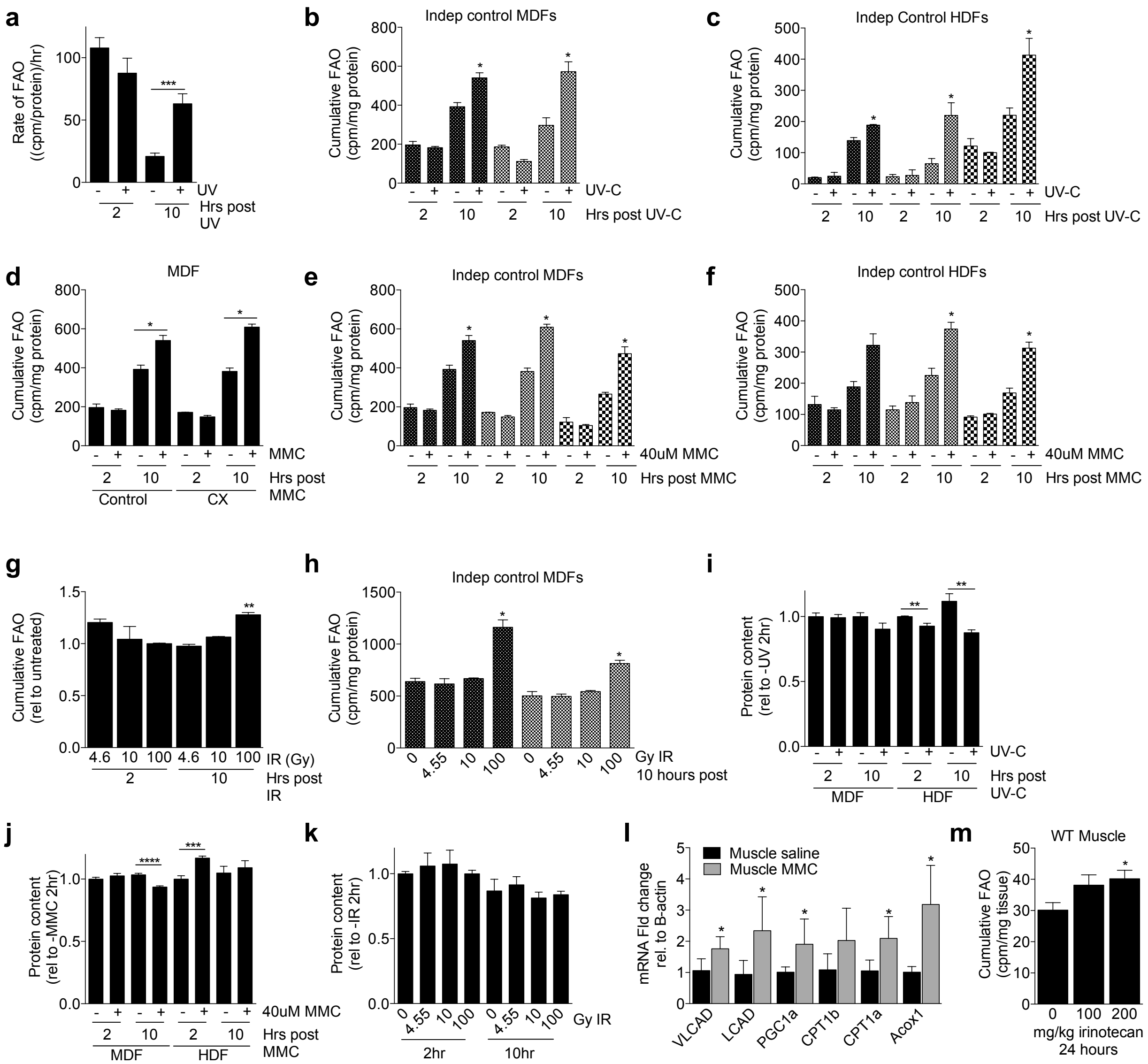

S4: Increased FAO is a general response to acute genotoxic stress. **a.** The rate of FAO of tritiated palmitate in WT MDFs between 2 and 10 hours after 20J/m^2 UV-C as described in Figure 4a; Student's T test. **b.** Two independent control MDF lines exposed to 20J/m^2 UV-C as described in Figure 4b; Student's T test between treatment groups within the indicated time point. **c.** Three independent WT HDF lines exposed to 24J/m^2 UV-C as described in Figure 4b, Student's T test between treatment groups within the indicated time point. **d.** Cumulative FAO of tritiated palmitate over the indicated time period of representative Control or CX MDFs exposed to 40μM MMC; Student's T test between treatment groups within time point. **e.** Three independent control MDF lines exposed to 40μM MMC as described in Figure 4d, Student's T test between treatment groups within the indicated time point. **f.** Three independent WT HDF lines exposed to 40μM MMC as described in Figure 4d, Student's T test between treatment groups within the indicated time point. **g.** Cumulative FAO of 2 independent MDF lines exposed to 4.6, 10 or 100Gy IR expressed relative to mock treated cells; Student's T test between indicted group and untreated. **h.** Two independent control MDF lines exposed to 0, 4.6, 10 or 100Gy IR as described in Figure S4g. **i**. Relative protein content of cells described in Figure 4a,b and Supplemental Figure 4a-c, n=3/group, Student's T test. **j.** Relative protein content of cells described in Figure 4d and Supplemental Figure 4e,f, n=3/group, Student's T test. **k.** Relative protein content of cells described in Supplemental Figure 4g,h, n=3/group;

Student's T test. **l.** Gene expression of FAO-related genes in muscle from 8wk WT mice injected IP with 10mg/kg MMC or saline and harvested after 24hrs as in Figure 4e-f, n=4/group; Student's T test. **m.** FAO capacity of muscle from 8wk WT mice injected IP with 100 or 200mg/kg irinotecan or saline and harvested after 24hrs as in Figure 4g, n=4/group; Student's T test. *p<0.05, **p<0.01, ***p<0.001, ****p<0.0001.

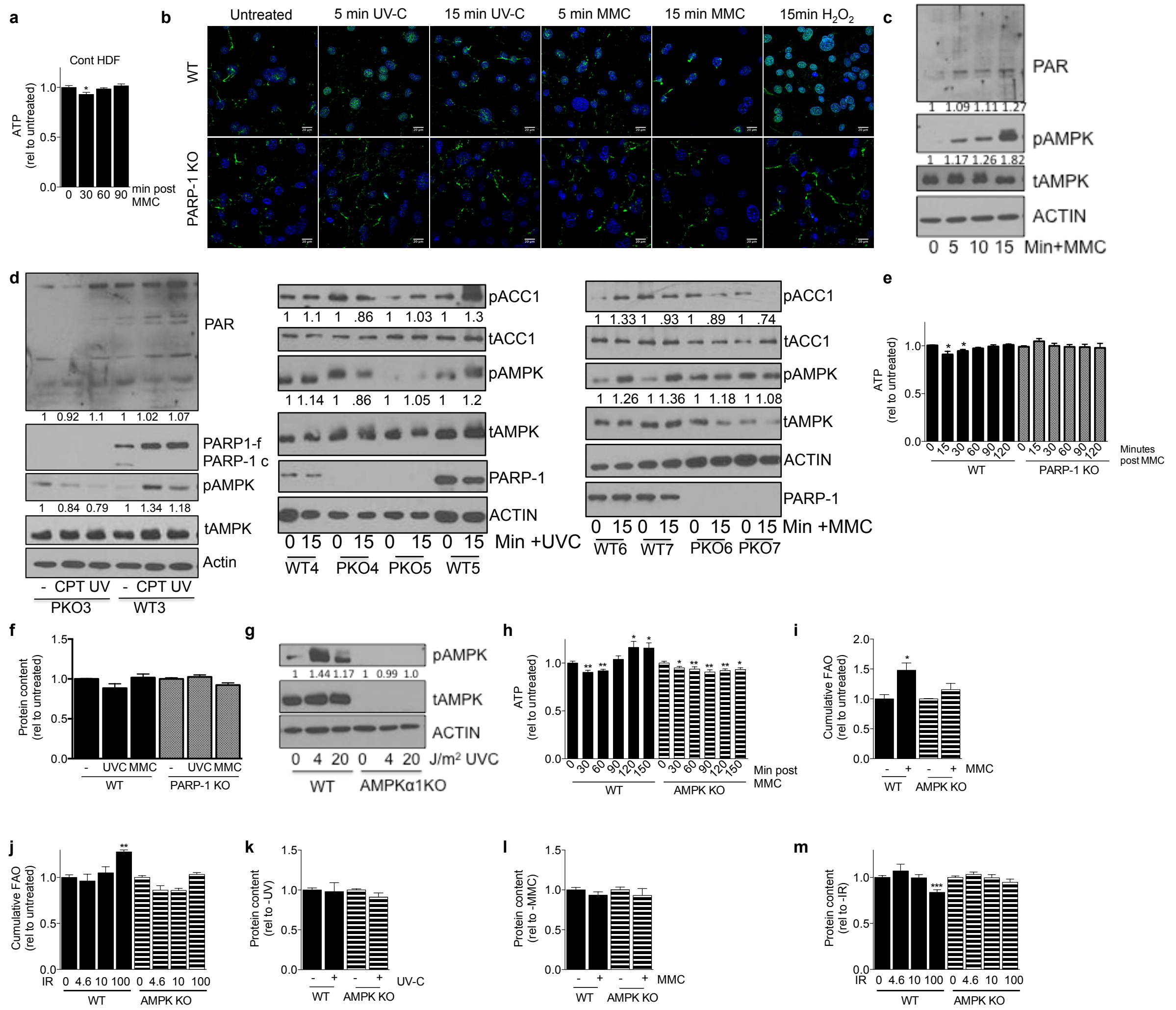

S5: PARP-1-dependent NAD+/ATP depletion and AMPK activation link DNA damage to increased FAO. a. Steady-state ATP levels of WT HDFs treated with 40μM MMC for the indicated time, n=4 lines/genotype; 1way ANOVA with Dunnett's multiple comparisons test. **b.** Immunofluorescence for PAR/DAPI in WT and PARP-1 KO MDFs 5 and 15 minutes after exposure to $20J/m^2$ UV-C, 40μM MMC, or 100μM H_2O_2. **c.** Western blot of WT MDFs 0, 5, 10, and 15 minutes after exposure to 40μM MMC. **d.** Western blots of five independent WT and PARP-1 KO MDF lines after 15-minute exposure to 100μM Camptothecin, 40μM MMC, or $20J/m^2$ UV-C as indicated. **e.** Steady-state ATP levels of WT and PARP-1 KO MDFs over a time course after exposure to 40μM MMC, n=3 lines/genotype; 1way ANOVA with Dunnett's multiple comparisons test. **f.** Relative protein content of WT and PARP-1 KO MDFs after exposures described in Figure 5e, n=3-4 lines/genotype. **g.** Western blot of WT and AMPKα1KO MDFs exposed to 1hr of 4 or $20J/m^2$ UV-C. **h.** Steady-state ATP levels of WT and AMPKα1KO MDFs over a time course after exposure to 40μM MMC, n=3-4 lines/genotype; 1way ANOVA with Dunnett's multiple comparisons test. **i.** Relative cumulative FAO of tritiated palmitate in WT and AMPKα1KO MDFs 10hrs after exposure to 40μM MMC, n=2 lines/genotype in duplicate; Student's T test relative to untreated within genotype. **j.** Relative cumulative FAO of tritiated palmitate in WT and AMPKα1KO MDFs 10hrs after exposure to 4.6, 10 and 100Gy ionizing radiation, n=2-4 lines/genotype in duplicate; Student's T test relative to untreated within genotype. **k-m.** Relative protein content of WT and AMPKα1KO MDFs after exposures described in Figure 5h, S5i, and S5j, respectively, n=2-4/group, Student's T test, *p<0.05, **p<0.01, ***p<0.001.

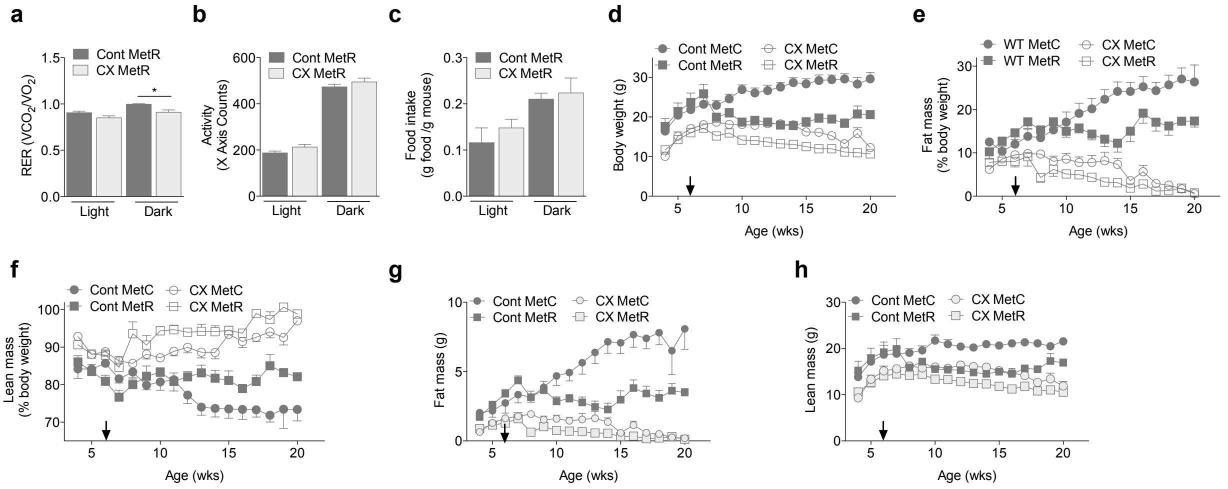

S6: Detrimental and beneficial adaptations of modulating fatty acid oxidation in response to genotoxic stress. RER **(a)**, horizontal activity **(b)**, and food consumption **(c)** of 10-14wk Control and CX mice fed a MetR diet for 2wks prior to indirect calorimetry, n=4/group; Student's T test. Body weight, **(d)** Percent fat **(e)** and lean **(f),** absolute fat **(g)** and lean **(h)** mass of Control and CX mice on MetC or MetR diets for 16wks, n=8-17/group, also shown in Figure 6c. $^*p<0.05$.
